# Our Contemporaries

**Published:** 1911-11

**Authors:** 


### OUR CONTEMPORARIES

THE *Practical Medicine,* edited by Ram Narain Mathur and published at Delhi, India, is one of our interesting exchanges that must be a blessing to a home-sick American doctor. The editor abstracts extensively from American journals, publishes original articles from an occasional American contributor and even the advertisements remind us of home.

DR. HENRY R. HARROWER, of Chicago, editor of *Physiologic Therapeutics,* published bi-monthly, has recently filled in the chinks with another bi-monthly, named *Successful Medicine.* This will be devoted to the business aspects of medical practice and, like Harrower's other undertakings, will be itself successful because of its merit.

WHAT others think of us. The *Maryland Medical Journal* for October reprints, with due acknowledgement, from our September issue, "The Control Experiment and the Enthusiasts," "Quality, not Quantity in Medical Education" and "The Splitting of Fees." The first and last were contributed articles. The *Journal of Ophthalmology and Oto-laryngology* has asked permission to reprint Dr. F. Parke Lewis's article, from the same issue—a permission granted with pleasure.

THE *Buffalo Commercial* celebrated its hundredth anniversary in October, with a large, well illustrated edition of unusual interest.

